# Alzheimer-like brain metabolic and structural features in cholesterol-fed rabbit detected by magnetic resonance imaging

**DOI:** 10.1186/s12944-018-0705-9

**Published:** 2018-03-27

**Authors:** Ping Jin, Yongming Pan, Zhiyong Pan, Jianqin Xu, Min Lin, Zhichao Sun, Minli Chen, Maosheng Xu

**Affiliations:** 1 The First Affiliated Hospital of Zhejiang Chinese Medical University, No. 54 Youdian Road, Shangcheng District, Hangzhou, Zhejiang 310006 People’s Republic of China; 2 The Third Affiliated Hospital of Zhejiang Chinese Medical University, Hangzhou, Zhejiang China; 3 Laboratory Animal Research Center/Comparative Medical Research Institute, Zhejiang Chinese Medical University, No 548 Binwen Road, Binjiang District, Hangzhou, 310053 China

**Keywords:** Cholesterol, Amyloid β, Rabbit, Magnetic resonance imaging, Magnetic resonance spectroscopy, Brain metabolite

## Abstract

**Background:**

Hypercholesterolemia is known to increase the risk of AD in later life, the purpose of this study is to illustrate brain metabolic and structural changes in a cholesterol-fed rabbit model of Alzheimer’s Disease (AD) by using clinical 3 T Magnetic Resonance Imaging (MRI).

**Methods:**

The Institutional Animal Care and Use Committee of Zhejiang Chinese Medical University approved the study. Totally 16 Japanese White Rabbits (JWR) were randomly divided into 2 groups including normal control group fed with routine diet (group NC) and high cholesterol diet group (group CD) fed a 2% cholesterol diet with 0.24 ppm copper in the drinking water for 12 weeks. Magnetic resonance spectroscopy (MRS) and structural image of rabbit brain were performed by using a 3 Tesla (T) MRI scanner with an 8 channel Rabbit coil. The chemical metabolites were identified by LC Model including N-acetylaspartate (NAA), creatine (Cr), glutamate (Glu), glutamine (Gln), Glycerophosphatidylcholine (GPC), phosphorylcholine (PCH), and myoinositol (MI). The relative concentrations (/Cr) were analyzed. Additionally, Amyloid-β (Aβ) accumulation in the brain was measured postmortem. For comparisons of MR and Aβ data between groups, two-tailed t-tests were performed.

**Results:**

The ratio of NAA/Cr (0.76 ± 0.10) and Glu/Cr (0.90 ± 0.14) in group CD were lower than those in the group NC (0.87 ± 0.06, 1.13 ± 0.22, respectively, *P* <  0.05). Compared to the group NC (2.88 ± 0.09 cm^3^, 0.63 ± 0.08 cm^3^, respectively), the cortical and hippocampal volumes (2.60 ± 0.14 cm^3^ and 0.47 ± 0.07 cm^3^, respectively) of rabbits brain decreased in the group CD while the third and lateral ventricular volumes enlarged (44.56 ± 6.01 mm^3^ vs 31.40 ± 6.14 mm^3^, 261.40 ± 30.98 mm^3^ vs 153.81 ± 30.08 mm^3^, *P* <  0.05). These metabolic and structural changes were additionally accompanied by the significant increase of Aβ1–42 in the cortex and hippocampus (163.60 ± 16.26 pg/mg and 215.20 ± 69.86 pg/mg, respectively, *P* <  0.05).

**Conclusion:**

High cholesterol diet can induce the brain metabolic and structural changes of the rabbit including lowered level of NAA and Glu and the atrophy of the brain which were similar to those of human AD.

## Background

Alzheimer’s disease (AD), the most common form of dementia, is characterized by the accumulation of β-amyloid (Aβ) plaques and neurofibrillary tangles (NFT) composed of tau amyloid fibrils [1]. AD is commonly associated with loss of neural synapse [2] and neurodegeneration leading to memory impairment and other cognitive ailments. Epidemiological studies have revealed the development of AD cannot be traced to a single root cause [3] and no known effective treatment seems to markedly slow the progression of AD [4]. As the processing of profound studies on the role of diet and nutraceuticals, the association of the high cholesterol diet or hypercholesterolemia with neurocognitive dysfunction, cardiovascular and other metabolic diseases has been reported [5, 6]. A growing body of evidence supports the claim that cholesterol metabolism plays a leading role in AD pathogenesis [7–9]. In addition to participating in the formation and degradation of Aβ, Cholesterol also modulates the function and aggregation of Aβ and influences its neurotoxic effects [10–12]. Owing to this, many cholesterol-fed animal models of AD have been established to study the pathophysiology of AD [13–15].

The hallmark of AD on magnetic resonance imaging (MRI) is hippocampal atrophy [16–18]. The workup of AD relies heavily on magnetic resonance imaging (MRI) which not only provides quantitative assessment of neural atrophy via structural magnetic resonance imaging (sMRI), but also detects changes of brain metabolism by proton magnetic resonance spectroscopy (^1^H MRS) [19, 20]. As a technique that can evaluate brain metabolites in living individuals non-invasively, ^1^HMRS has been used to assess neurodegenerative diseases through N-acetylasparate (NAA), glutamate (Glu), glutamine (Gln), myoinositol (MI), choline (Cho) levels. NAA levels are correlated to the regeneration of neurons, while MI is an indicator of gliocyte activity. Glu is the main excitatory neurotransmitter in the central nervous system and Cho acts as an indicator of cell membrane metabolism.

The goal of this study was to report the brain metabolic alterations of the cholesterol-fed JWR rabbit by MRS and show its brain structural changes via sMRI. Furthermore, we planned to explore the correlation between the Aβ accumulation and the data of brain metabolism and structure.

## Methods

### Animal

A total of 16 male JWR rabbits 3–4 months of age and weighting approximately 2 kg upon arrival were housed individually, with free access to rabbit chow and water. Animals were maintained on a 12-h light/12-h dark cycle and experiments were performed according to the Guidelines proposed by the Laboratory Animal Research Center of Zhejiang Chinese Medical University (SYXK, Hangzhou, 2013–0164, China); approved by the Institutional Animal Care and Use Committee of the Zhejiang Chinese Medical University. 8 JWR were fed 120 g/day of rabbit chow supplemented with 2% cholesterol. Eight additional age-matched rabbits were fed routine diet and served as controls. Rabbits were kept on their respective diets for 12 weeks.

### Blood biochemical index measurement

After an abrosia period of 12 h, blood samples were drawn from the JWR rabbits via the Auricular marginal veins. The serum total cholesterol (TC), high-density lipoprotein cholesterol (HDL-C), low-density lipoprotein cholesterol (LDL-C) and Triglycerides (TG) levels were analyzed by an auto-biochemical analyzer (7020, ITACHI, Japan) using the corresponding kits (Shanghai Shenneng-DiaSys Diagnostic Technology Co., Ltd., China).

### MRI data acquisition

**Fig. 1:**
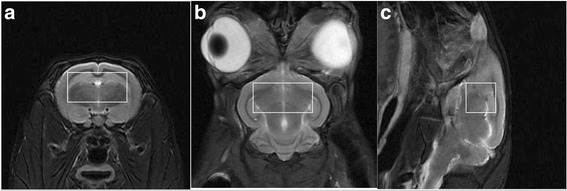
The region of interesting in MRS. The region of interesting was selected on the axial (**a**), coronary (**b**) and sagittal (**c**) T2W imaging of a rabbit brain with routine diet

MRI was performed before histological analysis. All rabbits were anesthetized by using 30 mg/kg ketamine and 4 mg/kg of xylazine injected intramuscularly 15 min before MRI. A 3 Tesla MRI scanner (GE Discovery MR750) was used with an 8 channels Rabbit coil (Shanghai Chenguang Medical Technologies Company, China). Rabbits were scanned in the prone position with the rabbit’s body extended and the rabbit’s head positioned in center of the magnetic field so as to obtain better signal noise ratio. The rabbits underwent following MRI scans: 1) 3-plane localizer scan to check animal positioning. 2) an axial T1-weighted 3D inversion-recovery spoiled gradient echo sequence (3D T1SPGR) for anatomical scanning with good differentiation between gray and white matter; 3) The axial, sagittal and coronary T2-weighted fast spin-echo sequence (FSE) to position the voxel of Magnetic Resonance Spectrum (MRS). 4) ^1^H MRS. 3D-T1 FSPGR by using TR = 7.8 ms, TE = Minimum ms, FOV = 100 × 100 mm and slice thickness = 1 mm. Matrix = 224 × 224, NEX = 1; Axial T2WI: TR 5500 ms, TE = 100 ms, slice thickness = 3 mm, space = 1 mm, FOV = 100 × 100 mm, Matrix = 352 × 256, NEX = 2. Coronal T2W FSE: TR 5500 ms, TE = 100 ms, slice thickness = 3 mm, space = 1 mm, FOV = 100 × 100 mm, Matrix = 352 × 224, NEX = 2. Sagittal T2W FSE: TR 4000 ms, TE = 75.8 ms, slice thickness = 3 mm, space = 1 mm, FOV = 100 × 100 mm, Matrix = 352 × 256, NEX = 2. ^1^H MRS scanning used point resolved spectroscopy (PRESS), TR = 2000 ms, TE = 35 ms, Voxel = 9 mm × 9 mm × 9 mm, total number of scans = 128, NEX = 8. The voxel was positioned in right brain which included parts of cortex, hippocampus and thalamus based on the axial, sagittal and coronary T2W (Fig. 1). Pre-scan, automatic shimming and water suppression were done before ^1^H-MRS was performed. The effect of Shimming was expressed as full width at half maximum (FWHM), if the FWHM was above 20 Hz, manual shimming would be employed for getting well homogeneity of magnetic field.

### MRS and sMRI analyses

The spectra of the routine diet and 12-week cholesterol diet rabbits were analyzed with LC Model (Version 6.3, Provencher SW.) [21]. The metabolites were identified including N-acetylaspartate (NAA), creatine (Cr), glutamate (Glu), Glycerophosphatidylcholine (GPC), phosphorylcholine (PCH), and myoinositol (MI). Well fitted metabolic peaks were included in the analyses when LC Model reported Cramer-Rao minimum variance bounds of less than 20%. The relative neurochemical concentrations (/Cr) including ratios of NAA/Cr, Glu/Cr, MI/ Cr and (GPC + PCH)/Cr were conducted automatically.

**Fig. 2:**
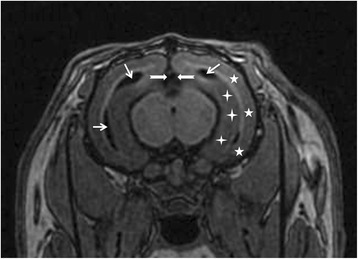
T1W image from a rabbit received high-cholesterol diet. The cortex (white pentagram), hippocampus (white cross), the third ventricles (white dovetail arrow) and lateral ventricles (white arrow) were identified

The cortex, hippocampus and ventricles of the rabbits were identified with reference to the rabbit brain atlas [22–24] (Fig. 2). The morphological measurement of the cortex and hippocampus were performed on the 3D SPGR images. Once the whole region of cortex or hippocampus was identified manually, the volume was calculated automatically with a standard volume measurement tool equipped in the workstation of MR system (ADW 4.6, GE, USA). Due to the thinner and irregular shape, the third and lateral ventricular volumes were calculated by multiplying the areas of each slice by the thickness of slice, and summing the volumes from each slice, And the areas of each slice was measured manually with a distance measurement tool in the ADW4.6 workstation of MRI. The atrophy rates of the cortex and hippocampus and the enlargement rates of the third and lateral ventricles were calculated.

### Aβ 1–42 level of cortex and hippocampus

Animals were anesthetized using 3% pentobarbital sodium 30 mg/kg injected via the marginal ear vein. The cortex and hippocampus of the rabbits were separated after the brains were extracted. A 10% *w*/*v* rabbit cortex and hippocampus homogenate were made in pre-chilled physiological saline using an IKA ULTRA-TURRAX homogenizer (IKA®-werke Gmbh & Co., KG, German), the obtained homogenate was centrifuged at 4500 rpm, 4 °C for 10 min. The supernatant was extract and the Aβ1–42 level was assayed immediately using homologous specific ELISA kits (Nanjing Jiancheng Bioengineering Institute, China), according to the manufacturer’s instructions. Protein concentration was determined by Coomassie brilliant blue method (Nanjing Jiancheng Bioengineering Institute, China).

### Statistical analysis

For comparisons of MR and Aβ data between cholesterol-fed and control group two-tailed t-tests were performed. The correlation between the Aβ accumulation and the data of brain metabolism and structure were explored by using Pearson correlation analyses. For all tests, the nominal level of significance was *P* <  0.05. GraphPad Prism 4.0a (GraphPad Software Inc., San Diego, CA) was used for statistical analysis.

## Results

### General characteristics of two JWR rabbits groups

Eight JWR were fed a 2% cholesterol diet for 12 weeks to establish an AD model. Compared to the NC group, cholesterol-fed animals in CD group had significantly higher TC, HDL-C and LDL-C levels (*P* <  0.01, Table 1).

**Table 1:** Phenotypic characteristics of WHBE rabbits fed a routine or cholesterol diet

Parameters	RD group (*n* = 8)	CD group (*n* = 8)	*P*-value
TC (mmol/L)	2.76 ± 0.71	48.69 ± 12.68	< 0.01
TG(mmol/L)	0.59 ± 0.09	2.06 ± 0.89	< 0.01
HDL-C(mmol/L)	0.73 ± 0.14	2.98 ± 0.29	< 0.01
LDL-C(mmol/L)	1.62 ± 0.57	32.89 ± 6.28	< 0.01

Abbreviations: *TC* total cholesterol, *TG* triglyceride, *HDL-C* high-density lipoprotein cholesterol, *LDL-C* low-density lipoprotein cholesterol, Values are shown as mean ± SD

### The metabolites in MRS

**Fig. 3:**
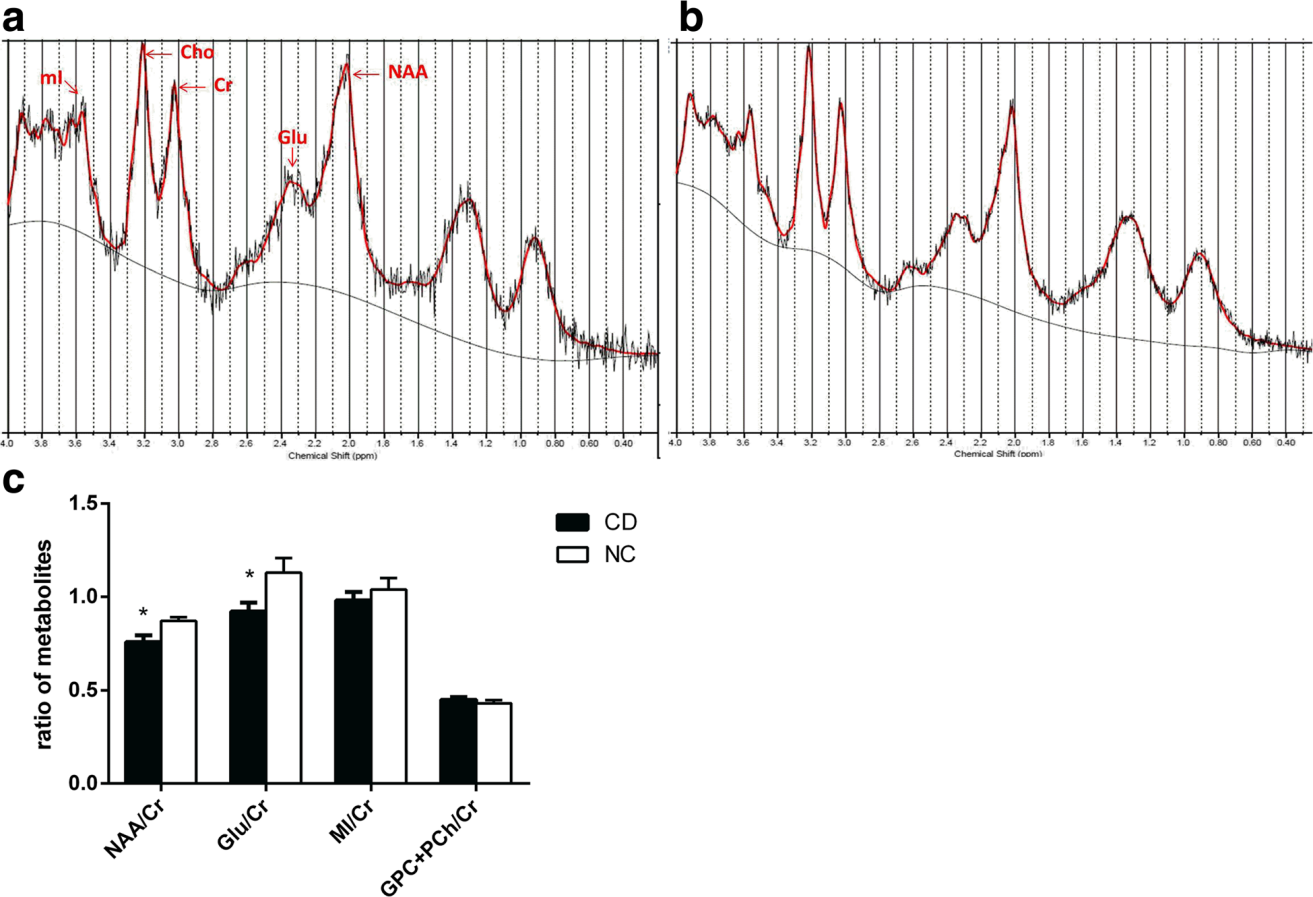
Mean (±SD) ratios of rabbit brain metabolites quantified by LCModel. The MRS of rabbit brain in the group RD (**a**) and group CD (**b**). The area under the metabolite peaks of the metabolites in the diagram represents the concentration of metabolites measured via LC model. Cr, creatine; Glu, glutamate; MI, myoinositol; NAA, N-acetylaspartate; PCh, phosphorylcholine; GPC, glycerophosphatidylcholine. **c** Mean (±SD) ratio of rabbit brain metabolites in two groups rabbits * *p* < 0.05. group NC Japanese white rabbits received routine diet (*n* = 8); Group CD, rabbits with routine diet plus 2% cholesterol (*n* = 8)

The values of NAA/Cr (0.76 ± 0.10) and Glu/Cr (0.90 ± 0.14) in high cholesterol diet (CD) group were lower than those in the NC group (0.87 ± 0.06, 1.13 ± 0.22, respectively, *P* <  0.05). On the other hand, there were no statistical significance on the values of MI/Cr, and (GPC + PCH)/Cr between two groups (Fig. 3).

### Structure of rabbit brain

**Fig. 4:**
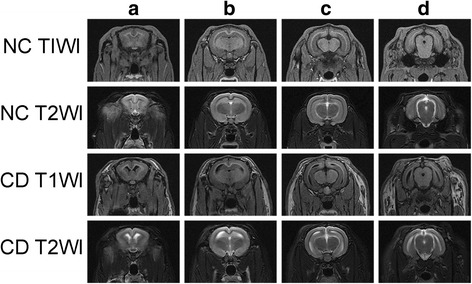
MRI of rabbit brain in two groups. T1W and T2W images on rabbit brain of two groups at different levels from left to right: the level of the rostral part of the hypophysis (**a**)*,* the caudal part of the hypophysis (**b**), the thalamus (**c**), and the mesencephalic aqueduct (**d**)

**Fig. 5:**
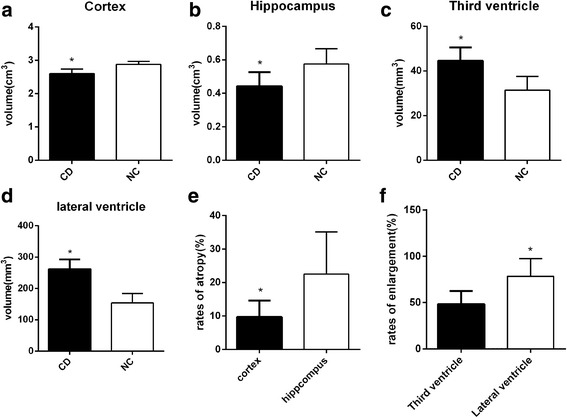
The changes of the structures in the brain. The volumes of cortex (**a**), hippocampus (**b**), third (**c**) and lateral ventricle (**d**) of two groups of rabbits. The atrophy rates of the cortex and hippocampus (**e**) and the enlargement rates of the third and lateral ventricles (**f**) JWR received routine diet (group NC, *n* = 8), rabbits with routine diet plus 2% cholesterol (group CD, *n* = 8). * *p* < 0.05

Compared to the group NC (2.88 ± 0.09 cm^3^, 0.57 ± 0.04 cm^3^, respectively), the cortical and hippocampal volumes (2.60 ± 0.14 cm^3^ and 0.44 ± 0.03 cm^3^, respectively) of rabbits in the group CD decreased, the third and lateral ventricular volumes enlarged (44.56 ± 6.01 mm^3^ vs 31.40 ± 6.14 mm^3^, 261.40 ± 30.98 mm^3^ vs 153.81 ± 30.08 mm^3^, *P* <  0.05) (Figs. 4 and 5). The atrophy of the hippocampus in cholesterol-fed rabbits is more evident than the cortex((22.51 ± 4.46)% vs (9.69 ± 1.72)%, *P* <  0.05), and the enlargement of the lateral ventricle is more evident than the third ventricle((78.44 ± 18.95)% vs (48.30 ± 14.22)%, *P* < 0.05).

### Aβ 1–42 levels in cortex and Hippocampus

**Fig. 6:**
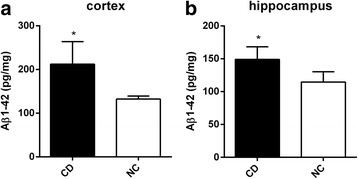
Mean (±SD) Aβ1–42 concentration. The Aβ1–42 concentration of the cortex (**a**) and hippocampus (**b**) of the rabbits with routine diet (group NC, *n* = 8) and rabbits with routine diet plus 2% cholesterol (group CD, *n* = 8). * *p* < 0.05

**Fig. 7:**
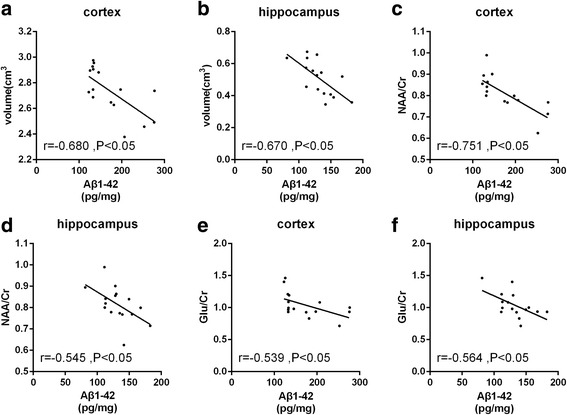
The correlations between the Aβ 1–42 and the NAA/Cr, Glu/Cr, cortical volume and hippocampal volume. The correlations between the Aβ 1–42 concentration of the cortex and the cortical volume (**a**),between the Aβ 1–42 concentration of the hippocampus and the hippocampal volume (**b**), between the Aβ 1–42 concentration of the cortex and the values of NAA/Cr (**c**), between the Aβ 1–42 concentration of the hippocampus and the values of NAA/Cr (**d**), between the Aβ 1–42 concentration of the cortex and the values of Glu /Cr (**e**) and between the Aβ 1–42 concentration of the hippocampus and the values of Glu /Cr (**f**)

The Aβ 1–42 levels differed between group CD and group NC. Compared to group NC, rabbits of group CD had higher cortical and hippocampal Aβ 1–42 levels (149.60 ± 16.26 pg/mg vs 114.5 ± 5.86 pg/mg and 212.20 ± 18.86 pg/mg vs 132.3 ± 2.40, respectively, *P* < 0.05) (Fig. 6). The Aβ 1–42 levels of cortex and hippocampus had significant correlations with the values of NAA/Cr and Glu/Cr and the volumes of the cortex and hippocampus (Fig. 7).

## Discussion

While many causes of AD are known, hypercholesterolemia is specifically known to drastically increase the risk of AD in later life. In our study we have established a cholesterol-fed rabbit model with significantly higher TC, TG, HDL-C and LDL-C levels. Furthermore, compared to the group NC, these rabbits in group NC were characterized by the significant increase of Aβ1–42 in the cortex and hippocampus. As the previous studies indicated [7–9], cholesterol metabolism plays a leading role in AD pathogenesis. Increased cholesterol levels can enhance the β-secretase pathway of APP to promote the formation of Aβ via lipid rafts [25] and reduce the degradation of Aβ by affecting the function of enzymes participating in the regulation of Aβ. These enzymes include insulin degrading enzyme (IDE) [26]. In addition, cholesterol also modulates the function and aggregation of Aβ and influences its neurotoxic effects [11].

We detected the variation of brain metabolites with 3.0 T MR Scanner. The ratio of NAA/Cr and Glu/Cr in cholesterol-fed JWR rabbits in the current study was significantly lower than the control group. This finding parallels previous clinical data regarding markers of AD which have reported that the ratio of NAA/Cr and Glu/Cr significantly decreases in AD patients compared to normal individuals [27, 28]. The ratio of NAA/Cr and Glu/Cr in our result is also in accord with the previous in vitro ^1^H MRS study in rats and mouse model of AD [29].

NAA mainly exists in neurons and is synthesized by chondriosome from acetyl-coenzyme A and aspartate. Although NAA remains a mysterious molecule, it is considered as a marker of neuronal vigour and number, the decreased level of NAA in MRS can be used as a representation of neuronal dysfunction or death [30].

Glu is the most abundant amino acids in mammalian brain. Converted from glutamine by neuron, Owe to the decreased level of Glu reflects the destruction of glutamatergic synapses in AD, some researchers pointed out the Glu may be more significant than NAA/Cr alone in representing the progress of AD [31, 32].

As the main excitatory neurotransmitter in the central nervous system, Glu could selectively active the N-methyl-D-asperate receptor (NMDAR), which is widely distributed in the brain. NMDAR is one of the most important excitatory amino acids receptor, and of great significance in the physiology and disease states. On the other hand, via upregulation of NMDAR, the excess Glu can causes the Ca^2+^ influx and induce excitatory neural toxicity leading to apoptosis [33]. As a previous study [34] showed indicated, cholesterol could increase the activity of NMDAR to intensify the neuronal excitability of Glu. As mentioned above, cholesterol could induce neuronal apoptosis not only through Aβ but also via NMDAR.

MI exists only in gliocyte, and is used as a marker of gliocyte health, with the rise of MI levels often used as an indicator of gliocyte proliferation [35].The activation of gliocytes is highly involved in the pathogenesis of AD. Activated gliocytes secrete immune molecules and activate complements to trigger the immune inflammatory reaction, resulting in neuronal damage in AD [36, 37]. Researchers investigated the levels of MI/Cr in AD have demonstrated varied conclusions. While some researches have suggested the level of MI/Cr [38] increases during AD, more studies pointed out the difference was not statistically significant between AD and normal control group [39, 40]. Our study showed the (GPC + PCH)/Cr ratio was not statistically different between the two groups.

Another finding of our study was that JWR rabbits fed a 2% cholesterol diet showed significant cortical and hippocampal atrophy, in accordance with current clinical standards for AD [16–18, 41].Similar to previous studies based on New Zealand White rabbit model of AD [42], cholesterol-fed JWR rabbits provided support for one of the most consistent findings in AD—an ventricular enlargement. In our study, the atrophy of the hippocampus is more evident than the cortex in cholesterol-fed rabbits, and the enlargement of the lateral ventricle adjacent to the hippocampus is more evident than the third ventricle. These result implied that hippocampus is affected earlier than other parts of brain by the hyperlipidemia. This brain characteristic in cholesterol-fed rabbit was similar to that of AD [43, 44]. As the hypothesis posed by Smith describes [45], in AD patients, the atrophy resulted from the formation of NFT begins in the hippocampus. With the loss of the input from the projection neurons in the hippocampus, the activity of neurons in neocortex begins to decrease, resulting in the decrease of the local cerebral blood flow and the formation of NFT.

Besides verifying cortical and hippocampal atrophy with ventricular enlargement in JWR rabbits fed a 2% cholesterol diet, our study detected the change of brain metabolites in cholesterol-fed JWR rabbit model using MRS, adding metabolic evidence to the feasibility of the cholesterol-fed rabbit model of AD. Yet, this study is not without limitations. Firstly, this study did not include any behavioral tests to verify clinical symptoms of memory loss. Secondly, a test for tau protein, which is important in AD, was not included in our study.

## Conclusion

Rabbits fed with cholesterol diet displayed Alzheimer-like signs of AD development, not only the cortical and hippocampal atrophy with ventricular enlargement, but also the alterations of brain metabolites such as lowered ratios of NAA/Cr and Glu/Cr.

## Abbreviations

^1^H MRS: Proton magnetic resonance spectroscopy
AD: Alzheimer’s disease
Aβ: Amyloid β
CD: Cholesterol diet
Cho: Choline
Gln: Glutamine
Glu: Glutamate
HDL-C: High-density lipoprotein cholesterol
JWR: Japanese white rabbits
LDL-C: Low-density lipoprotein cholesterol
MI: Myoinositol
MRI: Magnetic resonance imaging
NAA: N-acetylasparate
NFT: Neurofibrillary tangles
NMDAR: N-methyl-D-asperate receptor
RD: Routine diet
TC: Total cholesterol
TG: Triglycerides

## References

[1] Acker CM, Forest SK, Zinkowski R, Davies P, d’Abramo C (2013). Sensitive quantitative assays for tau and phospho-tau in transgenic mouse models. Neurobiol Aging.

[2] Braak H, Braak E (1991). Neuropathological stageing of Alzheimer-related changes. Acta Neuropathol.

[3] Hickman RA, Faustin A, Wisniewski T (2016). Alzheimer disease and its growing epidemic: risk factors, biomarkers, and the urgent need for therapeutics. Neurol Clin.

[4] Barrera-Ocampo A, Lopera F (2016). Amyloid-beta immunotherapy: the hope for Alzheimer disease?. Colomb Médi Cm.

[5] Scicchitano P, Cameli M, Maiello M, Modesti PA, Muiesan ML, Novo S (2014). Nutraceuticals and dyslipidaemia: beyond the common therapeutics. J Funct Foods.

[6] Zhao M, Zhang P, Guo Y, Zhao H (2017). Effect of high-fat diet on cognitive functions. Food Sci.

[7] Ehehalt R, Keller P, Haass C, Thiele C, Simons K (2003). Amyloidogenic processing of the Alzheimer beta-amyloid precursor protein depends on lipid rafts. J Cell Biol.

[8] Pappolla MA, Bryant-Thomas TK, Herbert D, Pacheco J, Fabra GM, Manjon M (2003). Mild hypercholesterolemia is an early risk factor for the development of Alzheimer amyloid pathology. Neurology.

[9] Whitmer RA, Sidney S, Selby J, Johnston SC, Yaffe K (2005). Midlife cardiovascular risk factors and risk of dementia in late life. Neurology.

[10] Maulik M, Westaway D, Jhamandas JH, Kar S (2013). Role of cholesterol in APP metabolism and its significance in Alzheimer’s disease pathogenesis. Mol Neurobiol.

[11] Matsuzaki T, Sasaki K, Hata J, Hirakawa Y, Fujimi K, Ninomiya T (2012). Association of Alzheimer disease pathology with abnormal lipid metabolism: the Hisayama study. Neurology.

[12] Carr DB, Goate A, Phil D, Morris JC (1997). Current concepts in the pathogenesis of Alzheimer’s disease. Am J Med.

[13] Götz J, Ittner LM (2008). Animal models of Alzheimer's disease and frontotemporal dementia. Nat Rev Neurosci.

[14] Gallagher M, Rapp PR (1997). The use of animal models to study the effects of AGING on cognition. Annu Rev Psychol.

[15] Sparks DL, Friedland R, Petanceska S, Schreurs BG, Shi J, Perry G (2006). Trace copper levels in the drinking water, but not zinc or aluminum influence CNS Alzheimer-like pathology. J Nutr Health Aging.

[16] Sluimer JD, van der Flier WM, Karas GB, Fox NC, Scheltens P, Barkhof F (2008). Whole-brain atrophy rate and cognitive decline: longitudinal MR study of memory clinic patients. Radiology.

[17] Jagust W (2006). Positron emission tomography and magnetic resonance imaging in the diagnosis and prediction of dementia. Alzheimers Dement.

[18] Jack CJ, Shiung MM, Weigand SD, O'Brien PC, Gunter JL, Boeve BF (2005). Brain atrophy rates predict subsequent clinical conversion in normal elderly and amnestic MCI. Neurology.

[19] Sole AD, Malaspina S, Biasina AM (2016). Magnetic resonance imaging and positron emission tomography in the diagnosis of neurodegenerative dementias. Funct Neurol.

[20] Dona O, Thompson J, Druchok C (2016). Comprehensive review on magnetic resonance imaging in Alzheimer's disease. Crit Rev Biomed Eng.

[21] Stephen WPPD (1993). Estimation of metabolite concentrations from localized in vivo proton NMR spectra. Magn Reson Med.

[22] Osofsky A, Lecouteur RA, Vernau KM (2007). Functional neuroanatomy of the domestic rabbit (Oryctolagus cuniculus). Vet Clin North Am Exot AnimPract.

[23] Muñozmoreno E, Arbatplana A, Batalle D, Soria G, Illa M, Pratsgalino A (2012). A magnetic resonance image based atlas of the rabbit brain for automatic Parcellation. PLoS One.

[24] Müllhaupt D, Augsburger H, Schwarz A, Fischer G, Kircher P, Hatt JM (2015). Magnetic resonance imaging anatomy of the rabbit brain at 3T. Acta Vet Scand.

[25] Marquer C, Devauges V, Cossec JC, Liot G, Lécart S, Saudou F (2011). Local cholesterol increase triggers amyloid precursor protein-Bace1 clustering in lipid rafts and rapid endocytosis. FASEB J.

[26] Bulloj A, Leal MC, Surace EI, Zhang X, Xu H, Ledesma MD (2008). Detergent resistant membrane-associated IDE in brain tissue and cultured cells: relevance to Abeta and insulin degradation. Mol Neurodegener.

[27] Hattori N, Abe K, Sakoda S, Sawada T (2002). Proton MR spectroscopic study at 3 tesla on glutamate/glutamine in Alzheimer's disease. Neuroreport.

[28] Rupsingh R, Borrie M, Smith M, Wells JL, Bartha R (2009). Reduced hippocampal glutamate in Alzheimer disease. Neurobiol Aging.

[29] Yao JL, Chen YW, Pang L, Li HH, You KZ, Cao Z (2011). Early changes of hippocampal metabolic in animal model of Alzheimer disease measured with 9.4T MR spectroscopy. Chin J Med Imaging Technol.

[30] Moffett JR, Ross B, Arun P, Madhavarao CN, Namboodiri AM (2007). N -Acetylaspartate in the CNS: from neurodiagnostics to neurobiology. Prog Neurobiol.

[31] Bell KF, Bennett DA, Cuello AC (2007). Paradoxical upregulation of glutamatergic presynaptic boutons during mild cognitive impairment. J Neurosci Official J Soc Neurosci.

[32] Antuono PG, Jones JL, Wang Y, Li SJ (2001). Decreased glutamate + glutamine in Alzheimer's disease detected in vivo with (1)H-MRS at 0.5 T. Neurology.

[33] Croce N, Bernardini S, Di CS, Caltagirone C, Angelucci F (2013). Hydrochloric acid alters the effect of L-glutamic acid on cell viability in human neuroblastoma cell cultures. J Neurosci Methods.

[34] Frank C, Giammarioli AM, Pepponi R (2004). Cholesterol perturbing agents inhibit NMDA-dependent calcium influx in rat hippocampal primary culture. FEBS Lett.

[35] Brand A, Richterlandsberg C, Leibfritz D (1994). Multinuclear NMR studies on the energy metabolism of glial and neuronal cells. Dev Neurosci.

[36] Bianca VD, Dusi S, Bianchini E, Dal PI, Rossi F (1999). Beta-amyloid activates the O-2 forming NADPH oxidase in microglia, monocytes, and neutrophils. A possible inflammatory mechanism of neuronal damage in Alzheimer's disease. J Biol Chem.

[37] Webster SD, Yang AJ, Margol L, Garzonrodriguez W, Glabe CG, Tenner AJ (2000). Complement component C1q modulates the phagocytosis of Abeta by microglia. Exp Neurol.

[38] Schuff N, Meyerhoff DJ, Mueller S, Chao L, Sacrey DT, Laxer K (2006). N-acetylaspartate as a marker of neuronal injury in neurodegenerative disease. Adv Exp Med Biol.

[39] Foy CML, Daly EM, Glover A, O’Gorman R, Simmons A, DGM M (2011). Hippocampal proton MR spectroscopy in early Alzheimer’s disease and mild cognitive impairment. Brain Topogr.

[40] Dixon RM, Bradley KM, Budge MM, Styles P, Smith AD (2002). Longitudinal quantitative proton magnetic resonance spectroscopy of the hippocampus in Alzheimer's disease. Brain.

[41] Magisetty O, Dowlathabad MR, Raichurkar KP, Mannar SN (2015). First magenetic resonance imaging studies on aluminium maltolate-treated aged New Zealand rabbits: an Alzheimer's animal model. Psychogeriatrics.

[42] Deci S, Lemieux SK, Smithbell CA, Sparks DL, Schreurs BG (2012). Cholesterol increases ventricular volume in a rabbit model of Alzheimer’s disease. J Alzheimers Dis Jad.

[43] Karas GB, Scheltens P, Rombouts SARB, Visser PJ, Schijndel RAV, Fox NC (2004). Global and local gray matter loss in mild cognitive impairment and Alzheimer's disease. NeuroImage.

[44] Tapiola T, Pennanen C, Tapiola M, Tervo S, Kivipelto M, Hänninen T (2008). MRI of hippocampus and entorhinal cortex in mild cognitive impairment: a follow-up study. Neurobiol Aging.

[45] Smith AD (2002). Imaging the progression of Alzheimer pathology through the brain. Proc Natl Acad Sci.

