# Correction to: Alzheimer-like brain metabolic and structural features in cholesterol-fed rabbit detected by magnetic resonance imaging

**DOI:** 10.1186/s12944-018-0840-3

**Published:** 2018-08-29

**Authors:** Ping Jin, Yongming Pan, Zhiyong Pan, Jianqin Xu, Min Lin, Zhichao Sun, Maosheng Xu, Minli Chen

**Affiliations:** 1 The First Affiliated Hospital of Zhejiang Chinese Medical University, No. 54 Youdian Road, Shangcheng District, Hangzhou Zhejiang, 310006 People’s Republic of China; 2 The Third Affiliated Hospital of Zhejiang Chinese Medical University, Zhejiang, Hangzhou China; 3 Laboratory Animal Research Center/ Comparative Medical Research Institute, Zhejiang Chinese Medical University, No 548 Binwen Road, Binjiang District, Hangzhou, 310053 China

## Correction

Unfortunately, after publication of this article [1], it was noticed that the order of correspondence addresses was reversed. Maosheng Xu should be listed before Minli Chen. The correct order of correspondence can be seen in this correction article.

## References

[1] Jin P, Pan Y, Pan Z, Xu J, Lin M, Sun Z (2018). Alzheimer-like brain metabolic and structural features in cholesterol-fed rabbit detected by magnetic resonance imaging. Lipids Health Dis.

